# White matter hyperintensities: a marker for apathy in Parkinson’s disease without dementia?

**DOI:** 10.1002/acn3.51159

**Published:** 2020-08-28

**Authors:** Yu Zhang, Guo yong Zhang, Zi en Zhang, An qi He, Jing Gan, Zhenguo Liu

**Affiliations:** ^1^ Department of Neurology Xinhua Hospital Affiliated to Shanghai Jiao Tong University School of Medicine 1665 Kong jiang Road Shanghai 200092 People's Republic of China

## Abstract

**Objective:**

The objective of this study was to assess the relationship between white matter hyperintensities (WMH) and the occurrence and progression of apathy in Parkinson’s disease (PD).

**Methods:**

We recruited patients with PD who underwent baseline evaluation, which included apathy assessment and magnetic resonance imaging (MRI) head scans. After 2.5 years of follow‐up, we re‐evaluated patient apathy symptoms. The severity and location of WMH were assessed with fluid‐attenuated inversion recovery (FLAIR) sequences using the Fazekas visual rating scale. Logistic regression and linear regression analyses of baseline WMH characteristics were conducted to explore the potential association between apathy and WMH.

**Results:**

A total of 141 PD patients were recruited. The apathy group had a higher proportion of male patients, advanced disease, and depression, which was coupled with a lower quality of life. Morever, higher WMH severity was significantly associated with apathy. Logistic regression analyses demonstrated that WMH severity was a risk factor for apathy. In addition, linear regression analysis also suggests that apathy severity is positively correlated with baseline WMH Fazekas scales (ϐ = 0.959, *P* < 0.001). Baseline WMH severity was also a risk factor for apathy progression.

**Interpretation:**

WMH is associated with apathy and could be a promising marker to predict apathy progression in PD.

## Introduction

Apathy can be defined as a state of decreased motivation. It is characterized by reduced interests or negative emotions that cannot be attributed to a diminished level of consciousness, cognitive impairment, or emotional distress.^1^ The prevalence of apathy ranges from 13.9% to 70% in Parkinson’s disease (PD), and diagnosis is influenced by many factors, such as demographics and the diagnostic criteria and evaluation scales used.^2^ As a nonmotor symptom of PD, it significantly reduces the quality of life and places a burden on patients and their caregivers. ^3^ The risk factors for and pathogenesis of apathy are complicated and have not been clearly identified.

Recent MRI studies have shown that apathy is associated with spatially extensive reductions in white matter microstructural integrity. Anne et al^4^ found that apathy symptoms are associated with a loss of both gray and white matter volumes in elder Icelandic individuals without dementia. Claire et al^5^provided evidence that distinct structural network changes in white matter were associated with apathy using diffusion tensor imaging (DTI). Therefore, white matter changes appear to be an important apathy influencing factor. However, DTI is infrequently used clinically. White matter hyperintensities (WMH) and increased signals on fluid‐attenuated inversion recovery (FLAIR) brain image sequences are commonly present in patients with PD, and the severity and location of WMH can be easily evaluated using the Fazekas visual scale.^6^ However, there are a limited number of studies study the association between apathy and WMH in PD patients. In this study, we hypothesized that WMH might be a potential marker for apathy in PD patients.

## Patients and Methods

### Patients

**Figure 1:**
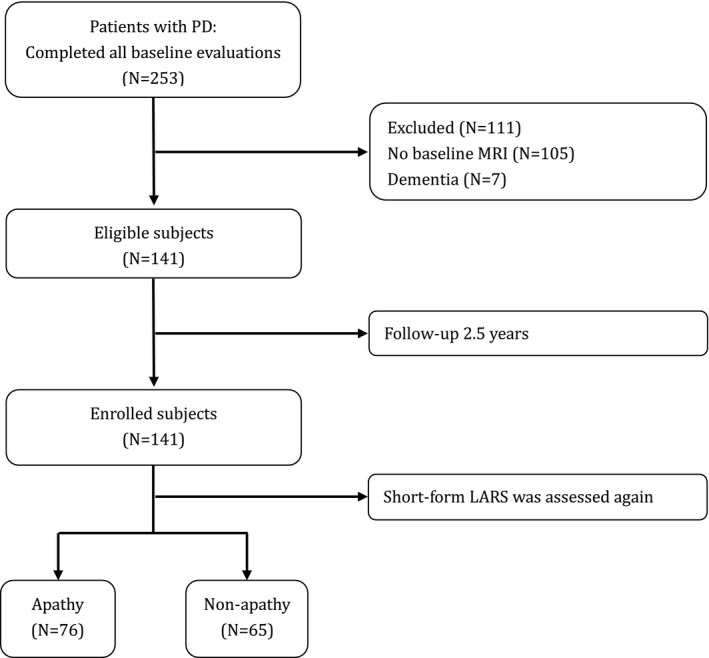
Flowchart of the enrollment of study participants.

All subjects were recruited from the Department of Neurology at Xin Hua Hospital affiliated with the Shanghai Jiao Tong University School of Medicine from December 2014 to April 2017. Patients were diagnosed with PD according to the Movement Disorder Society PD Criteria.^7^ All 253 patients with PD completed baseline evaluations, including demographic surveys and motor and nonmotor symptoms tests (see Supplementary Material S1). The following exclusion criteria were applied: (1) a baseline cranial MRI was not performed (n = 105) or (2) there was a diagnosis of dementia with PD (n = 7). Thus, a total of 141 patients without PD‐associated dementia were included in the final study population. The patients were followed up longitudinally, during which time they performed a short‐form LARS test. Written informed consent was obtained from all participants, and the study was performed with the approval of the Ethics Committee of Xin Hua Hospital affiliated with Shanghai Jiao Tong University School of Medicine. Figure 1 shows a flow chart of study participant enrollment.

### Clinical assessment

Demographic and clinical data were collected, which included gender, age, age of PD onset, predominant symptoms at PD onset, duration of disease, educational level, smoking and alcohol consumption histories, and dopamine replacement therapy and related complication histories. The total levodopa equivalent daily dose (LEDD) was calculated according to a previously suggested conversion formulae.^8^ The severity of motor symptoms was assessed with the Unified Parkinson’s Disease Rating Scale part III (UPDRS‐III) and Hoehn‐Yahr (H&Y) stage for each patient. The nonmotor symptoms were measured with the following scales: the scale for freezing of gait, the Minimum Mental State Examination (MMSE), the Parkinson's Disease Sleep Scale (PDSS), the Epworth Sleepiness Scale (ESS), the REM Sleep Behavior Disorder Questionnaire Hong Kong (RBDQ‐HK), the Fatigue Severity Scale (FSS), the nonmotor symptom(NMS) questionnaire, the Hamilton Depression Scale (HAMD), and the Parkinson’s Disease Questionaire‐39 (PDQ‐39).^9^, ^10^, ^11^, ^12^

Symptoms of apathy were assessed with the short‐form of the Lille Apathy Rating Scale (LARS).^13^ PD patients are considered to be suffering from apathy if they score >‐7, with a sensitivity of 87.50% and a specificity of 93.51%, according to earlier suggested cut‐offs.^13^

### Neuroimaging acquisition

**Figure 2:**
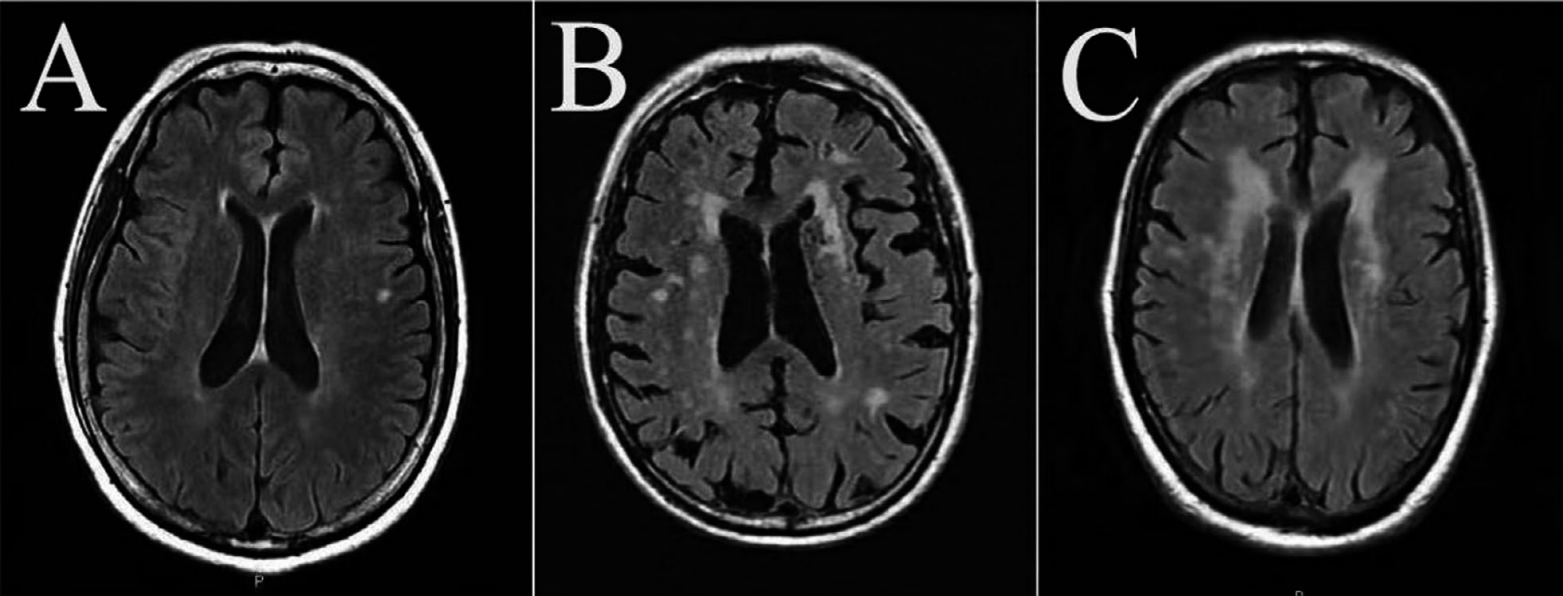
Representative T2‐FLAIR images of WMH A: PVH grade 1 and DWMH grade 1; B: PVH grade 2 and DWMH grade 2; C: PVH grade 3 and DWMH grade 3. Abbreviations: WMH, white matter hyperintensities; PVH, periventricular hyperintensities; DWMH, deep subcortical white matter hyperintensities.

A conventional head MRI at 3.0‐T (Sigma, GE Medical Systems, Milwaukee, WI, USA) was performed on 141 PD patients who were currently on medication. FLAIR images were used to grade WMH. FLAIR sequence images were acquired with the following parameters:turbo spin echo, repeat time (TR) = 8000 ms, echo time (TE) = 340 ms, T1 = 2400 ms, matrix = 256×256, slice thickness = 1 mm, 170 slices, and voxel size = 1.0 × 1.0 × 1.0 mm^3^. Head MRI scans were evaluated by two experienced neurologists who were blind to the clinical status of each patient. The Fazekas visual semiquantitative rating scale was used to easily assess the severity and location of WMH. The locations of WMH were divided into two different types: periventricular hyperintensities (PVH) and deep white matter hyperintensities (DWMH). PVH were graded as 0 = absence, 1 = “caps” or pencil‐thin lining, 2 = smooth “halo,” or 3 = irregular PVH extending into the deep white matter. DWMH were rated as 0 = absence, 1 = punctate foci, 2 = beginning confluence of foci, or 3 = large confluent areas ^[6]^.The Fazekas WMH score was the total of the deep and periventricular WMH scores (Figure 2).

### Statistical analysis

The characteristics of our study were summarized using means and standard deviations (SD) for continuous variables and frequencies and percentages (%) for categorical variables. The comparisons were performed by the Pearson’s Chi‐Square test for categorical variables. For continuous variables, Student's t‐tests were used to analyze variables with parametric distributions, and Mann–Whitney U tests were used to test those with nonparametric distributions. The differences in WMH locations in apathy and nonapathy groups were tested by a Chi‐Square test. Linear regression and logistic regression analyses were conducted to analyze the differences in WMH severity in apathy and non‐apathy groups. Logistic regression analyses were adjusted for gender, age, age at PD onset, disease duration, and educational level in model 1. In model 2, we additionally adjusted for smoking, alcohol consumption, initial presentation of motor symptoms, and total levodopa equivalent dose. Model 3 was additionally adjusted for Hoehn and Yahr stage, UPDRS part III, and PDSS score. The level of significance was set at *P* < 0.05. Odds ratios (ORs) were presented with their 95% confidence intervals (95% CI). We estimated the area under the receiver operating characteristic curve (AUC) to assess the ability of WMH to discriminate between apathy and non‐apathy states. Linear regression analysis and logistic regression analysis were conducted to analyze the association between WMH severity and apathy progression. For the statistical analysis and to generate graphs, SPSS‐24 and Prism 8.0 for Windows (GraphPad Software, Inc., San Diego, CA, USA) were used.

## Results

### Baseline demographic characteristics in PD patients with and without apathy

Pertinent details for total of 141 participants who underwent MRI scans (68 with apathy and 73 without) are presented in Table 1. There was a higher prevalence of males in the apathy versus the nonapathy group (63.24% vs. 45.21%, *P* = 0.032). The apathy group also had a higher Hoehn and Yahr stage on average (2.53 ± 0.65 vs. 2.08 ± 0.69, *P* < 0.001), higher UPDRS‐III scores(24.60 ± 13.25 vs. 17.73 ± 10.17, *P* = 0.001), higher depression scores(11.74 ± 7.64 vs. 7.56 ± 6.24, *P* = 0.001), higher NMS scores(41.34 ± 27.97 vs. 27.51 ± 21.62, *P* = 0.002), higher PDQ‐39 scores(27.10 ± 20.32 vs. 15.90 ± 12.86, *P* < 0.001), and higher WMH Fazekas scales(2.69 ± 1.33 vs. 1.66 ± 1.04, *P* < 0.001). In addition, the apathy group showed a tendency to be older in age (70.46 ± 7.55 years‐old vs. 68.01 ± 9.11 years‐old, *P* = 0.087) with a lower educational level (*P* = 0.054), lower PDSS scores (115.54 ± 21.02 vs. 121.89 ± 19.08, *P* = 0.050), and a higher frequency of freezing of gait (41.18% vs. 27.40%, *P* = 0.084). The following characteristics did not show significant differences between the two groups: age at disease onset, disease duration, MMSE scores, smoking frequency, alcohol use, medication use, initial presentation of motor symptoms, wearing off, dyskinesia, EDS, RBD, and fatigue.

**Table 1:** Baseline clinical characteristics of the patients in the apathy and nonapathy groups.

Variable	Total n = 141	Apathy n = 68	Non‐apathy n = 73	*P*‐value
Male gender, n (%)^1^	76 (53.90)	43 (63.24)	33 (45.21)	0.032*
Age (years)^2^	69.35 ± 8.42	70.46 ± 7.55	68.01 ± 9.11	0.087
Age at PD onset, (years)^3^	63.09 ± 9.11	63.71 ± 9.31	62.51 ± 8.95	0.235
Disease duration (years)^3^	6.18 ± 4.53	6.75 ± 5.44	5.64 ± 3.43	0.508
Educational level, n (%)^1^				0.054
None/first level, n (%)	13 (9.22)	10 (14.71)	3 (4.11)	
Secondary level/high school, n (%)	92 (65.25)	39 (57.35)	53 (72.60)	
University, n (%)	36 (25.53)	19 (27.94)	17 (23.29)	
Smoking, n (%)^1^	22 (15.60)	14 (20.59)	8 (10.96)	0.115
Alcohol, n (%)^1^	14 (9.93)	7 (10.29)	7 (9.59)	0.889
L‐dopa medication, n (%)^1^	115 (81.56)	56 (82.35)	59 (80.82)	0.815
L‐dopa LED, mg^2^	386.12 ± 346.87	419.63 ± 404.38	354.90 ± 282.38	0.535
DA medication, n (%)^1^	79 (56.03)	39 (57.35)	40 (54.79)	0.760
DA LED, mg^3^	45.12 ± 50.37	45.59 ± 49.61	44.69 ± 51.41	0.739
MAO‐B medication, n (%)^1^	33 (23.40)	15 (22.06)	18 (24.66)	0.716
MAO‐B LED, mg^3^	21.63 ± 65.00	14.34 ± 29.71	28.42 ± 85.42	0.640
Total LED, mg^3^	469.89 ± 384.07	501.61 ± 427.25	440.34 ± 339.31	0.550
Initial presentation of motor symptoms, n (%)^1^				0.914
Tremor	93 (65.96)	44 (64.71)	49 (67.12)	
Rigid	16 (11.35)	7 (10.29)	9 (12.33)	
Bradykinesia	22 (15.60)	12 (17.65)	10 (13.70)	
Other	10 (7.09)	5 (7.35%)	5 (6.85)	
Hoehn and Yahr stage^3^	2.29 ± 0.70	2.53 ± 0.65	2.08 ± 0.69	<0.001*
UPDRS part III^3^	21.04 ± 12.21	24.60 ± 13.25	17.73 ± 10.17	0.001*
Wearing off, n (%)^1^	57 (40.43)	28 (41.18)	29 (39.73)	0.861
Dyskinesia, n (%)^1^	15 (10.64)	8 (11.76)	7 (9.59)	0.675
Freezing of gait, n (%)^1^	48 (34.04)	28 (41.18)	20 (27.40)	0.084
MMSE score^3^	26.84 ± 3.03	26.31 ± 3.49	27.33 ± 2.44	0.140
HAMD‐24 score^3^	9.57 ± 7.23	11.74 ± 7.64	7.56 ± 6.24	0.001*
Depression, n (%)^1^	83 (58.87)	51 (75.00)	32 (43.84)	<0.001*
EDS, n (%)^1^	37 (26.24)	19 (27.94)	18 (24.66)	0.658
PDSS score^3^	118.83 ± 20.22	115.54 ± 21.02	121.89 ± 19.08	0.050
RBD, n (%)^1^	74 (52.48)	39 (57.35)	35 (47.95)	0.264
Fatigue, n (%)^1^	31 (21.99)	15 (22.06)	16 (21.92)	0.984
NMS score^3^	34.18 ± 25.75	41.34 ± 27.97	27.51 ± 21.62	0.002*
PDQ‐39 score^3^	21.30 ± 17.73	27.10 ± 20.32	15.90 ± 12.86	<0.001*
WMH Fazekas scale^3^	2.16 ± 1.29	2.69 ± 1.33	1.66 ± 1.04	<0.001*

Abbreviations: DA, Dopamine agonists; EDS, excessive daytime sleepiness; HAMD, Hamilton Depression Scale; LED, Levodopa Equivalent Dose; MAO‐B, Monoamine oxidase‐B; MMSE, Mini‐Mental State Examination; NMS, nonmotor symptom; PD, Parkinson’s disease; PDQ‐39, 39‐item Parkinson’s Disease Questionnaire; PDSS, Parkinson’s Disease Sleep Scale; RBD, Rapid Eye Movement Sleep Behavior Disorder; UPDRS, Unified Parkinson’s Disease Rating Scale; WMH, white matter hyperintensities.

^1^ Chi‐squared tests for categorical variables. Values are expressed as a number (percentage).

^2^ Student t tests for continuous variables with a parametric distribution.

^3^ Mann–Whitney U tests for continuous variables with a nonparametric distribution.

* Statistically significant (*P* < 0.05).

### Differences in WMH severity in PD patients with and without apathy

The WMH severity grade characteristics of 114 PD patients are summarized in Table 2. The apathy group had higher WMH Fazekas scales (2.69 ± 1.33 vs. 1.66 ± 1.04, *P* < 0.001). A chi‐square (χ^2^) test showed that the apathy status (presence or absence) was associated with the WMH severity grade. Furthermore, the p‐value for the linear‐by‐linear association revealed a trend where increasing WMH severity was associated with an increasing proportion of patients with apathy (*P* < 0.001). However, significant differences between depression and the severity of WMH were not observed. Overall, WMH severity was associated with apathy, but not depression in PD patients. Further analysis of the linear regression showed that short‐form LARS scores were positively correlated with WMH Fazekas scales (ϐ = 1.828, *P* < 0.001), DWMH grade (ϐ = 3.141, *P* < 0.001), and PVH grade (ϐ = 2.988, *P* < 0.001), while HAMD scores did not correlate with WMH Fazekas scales (ϐ = 0.462, *P* = 0.330), DWMH grade (ϐ = 0.774, *P* = 0.392) and PVH grade (ϐ = 0.778, *P* = 0.354).

**Table 2:** The characteristics of WMH severity in patients with apathy or depression.

Variable	Total n = 141	Apathy n = 68	Non‐apathy n = 73	*P*‐value	*P*‐value^3^	Depression n = 83	Non‐depression n = 58	*P*‐value	*P*‐value^3^
WMH Fazekas scale*, ^1^	2.16 ± 1.29	2.69 ± 1.33	1.66 ± 1.04	<0.001*	‐‐	2.28 ± 1.35	1.98 ± 1.21	0.164	‐‐
WMH grade^2^				<0.001*	<0.001*			0.194	0.072
Low, n (%)	35 (24.82)	8 (11.76)	27 (36.99)			17 (20.48)	18 (31.03)		
Moderate, n (%)	87 (61.70)	44 (64.71)	43 (58.90)			52 (62.65)	35 (60.34)		
High, n (%)	19 (13.48)	16 (23.53)	3 (4.11)			14 (20.59)	5 (8.62)		
PVH grade^2^				<0.001*	<0.001*			0.465	0.231
0, n (%)	17 (12.06)	1 (1.47)	16 (21.92)			8 (9.64)	9 (15.52)		
1, n (%)	87 (61.70)	39 (57.35)	48 (70.59)			51 (61.45)	36 (62.07)		
2‐3, n (%)	37 (26.24)	28 (41.18)	9 (13.24)			24 (28.92)	13 (22.41)		
DWMH grade^2^				<0.001*	<0.001*			0.497	0.240
0, n (%)	31 (21.99)	8 (11.76)	23 (33.82)			16 (19.28)	15 (25.86)		
1, n (%)	91 (64.54)	44 (64.71)	47 (69.12)			54 ( (65.06)	37 (63.79)		
2‐3, n (%)	19 (13.48)	16 (23.53)	3 (4.41)			13 (15.66)	6 (10.34)		

Abbreviations: 2–3 = moderate; and 4–6 = high; DWMH, deep subcortical white matter hyperintensities; PVH was graded from 0 to 3: 0 = absence, 1 = “caps” or pencil‐thin lining, 2 = smooth “halo,” and 3 = irregular PVH extending into the deep white matter. Separately, DWMH was graded from 0 to 3 as well: 0 = absence, 1 = punctate foci, 2 = beginning confluence of foci, and 3 = large confluent areas. The severity of the WMH was graded according to the sum of the score for PVH (0–3) and the score for DWMH (0–3): 0–1 = low; PVH, periventricular hyperintensities; WMH, white matter hyperintensities.

^1^ Mann–Whitney U tests for continuous variables with a nonparametric distribution.

^2^ Chi‐squared tests for categorical variables. Values are expressed as a number (percentage).

^3^ p for linear‐by‐linear association

* Statistically significant (*P* < 0.05).

We performed a Chi‐Square test to investigate how WMH location might differ in apathy. We found that there were no significant differences in the prevalence or absence of DWMH and PVH (DWMH and PVH grade was 0) between the apathy and nonapathy group (*P* = 0.131). There was also no significant difference between the depression and nondepression group in regards to the location of the WMH (*P* = 0.763). Overall, the WMH location was not associated with apathy or depression in PD.

### Independent association with WMH severity and apathy

The independent association between WMH severity and apathy is shown in Table 3. The apathy group had a significantly higher WMH severity compared to the nonapathy group. This association was attenuated somewhat after additional adjustment for covariates in models 2 and 3 but remained significant. The area under the ROC curve (AUC) for discriminating between the apathy and nonapathy group was the WMH Fazekas scale (0.722, 95% CI (0.639‐0.805)), followed by the PVH grade (0.707, 95% CI (0.622‐0.791)), and the DWMH grade (0.661, 95% CI (0.572‐0.750)),which demonstrated diagnostic value. The optimal cut‐off level for the total WMH score that discriminated between apathy and nonapathy groups was 2.5. Since the scoring system did not include 0.5, it could be inferred that a score of ≥3 was indicative of a diagnosis of apathy with a sensitivity of 42.6% and a specificity of 89.0%. However, depression was not associated with the severity of WMH, DWMH, or PVH in the logistic regression.

**Table 3:** Logistic regression analysis between WMH severity characteristics and apathy or depression according to different models.

Variable	Apathy						Depression					
	Model 1		Model 2		Model 3		Model 1		Model 2		Model 3	
	OR (95% CI)	*P*‐value	OR (95% CI)	*P*‐value	OR (95% CI)	*P*‐value	OR (95% CI)	*P*‐value	OR (95% CI)	*P*‐value	OR (95% CI)	*P*‐value
WMH Fazekas scale	2.223 (1.493‐3.310)	<0.001*	2.219 (1.480‐3.327)	<0.001*	2.143 (1.390‐3.303)	0.001*	1.187 (0.873‐1.614)	0.274	1.184 (0.863‐1.625)	0.296	1.075 (0.735‐1.572)	0.708
PVH grade	4.042 (2.037‐8.018)	<0.001*	3.877 (1.935‐7.765)	<0.001*	3.312 (1.608‐6.823)	0.001*	1.370 (0.800‐2.345)	0.251	1.443 (0.822‐2.531)	0.201	0.957 (0.478‐1.916)	0.957
DWMH grade	3.007 (1.555‐5.816)	0.001*	3.288 (1.646‐6.566)	0.001*	3.461 (1.636‐7.322)	0.001*	1.278 (0.718‐2.272)	0.404	1.203 (0.664‐2.179)	0.542	1.356 (0.659‐2.790)	0.409

Abbreviations: WMH, white matter hyperintensities; PVH, periventricular hyperintensities; DWMH, deep subcortical white matter hyperintensities; CI, confidence interval; UPDRS, Unified Parkinson’s Disease Rating Scale; MMSE, Mini‐Mental State Examination; PDSS, Parkinson’s Disease Sleep Scale.

Model 1: adjusting for gender, age, age at PD onset, disease duration, and educational level.

Model 2: adjusting for gender, age, age at PD onset, disease duration, educational level, smoking, alcohol, initial presentation of motor symptoms, and total levodopa equivalent dose.

Model 3: adjusting for gender, age, age at PD onset, disease duration, educational level, smoking, alcohol, initial presentation of motor symptoms, and total levodopa equivalent dose, Hoehn and Yahr stage, UPDRS part III, PDSS score.

*Statistically significant (*P* < 0.05).

### Differences in baseline data during apathy progression

After 2.5 years of follow‐up, we were able to measure apathy score changes by subtracting baseline scores from follow‐up scores (△short‐form LARS = follow‐up short‐form LARS － baseline short‐form LARS). Of the 114 PD patients, 76 (53.90%) were diagnosed with apathy after 2.5 years follow‐up. Patients were considered to suffer from apathy progression if △short‐form LARS>0. Apathy progression data is summarized in Table 4. Patients in the apathy progression group were older on average (71.97 ± 6.93 years vs. 66.45 ± 8.96 years, *P* < 0.001), had an older age of PD onset (65.37 ± 8.00 years vs. 60.83 ± 9.63, *P* = 0.001), and had higher WMH Fazekas scales (2.90 ± 1.31 vs. 1.42 ± 0.75, p＜0.001) versus patients not showing signs of progression. This group also had a tendency to present with a higher H&Y stage (2.41 ± 0.75) versus patients not showing apathy progression (2.18 ± 0.64, *P* = 0.069). The baseline short‐form LARS scores, disease duration, MMSE scores, smoking habits, alcohol consumption, wearing off, dyskinesia, EDS, RBD, and fatigue were not significantly different between the two groups.

**Table 4:** Baseline clinical characteristics of the patients with and without apathy progression.

Variable	Total n = 141	△short‐form LARS> 0 n = 70	△short‐form LARS ≤ 0 n = 71	*P*‐value
Male sex, n (%)^1^	76 (53.90)	41 (58.57)	35 (49.30)	0.269
Age (years)*, ^2^	69.35 ± 8.42	71.97 ± 6.93	66.45 ± 8.96	<0.001*
Age at PD onset, (years)^3^	63.09 ± 9.11	65.37 ± 8.00	60.83 ± 9.63	0.001*
Disease duration (years)^3^	6.18 ± 4.53	6.61 ± 4.98	5.75 ± 4.03	0.345
Hoehn and Yahr stage^3^	2.29 ± 0.70	2.41 ± 0.75	2.18 ± 0.64	0.069
Wearing off, n (%)^1^	57 (40.43)	33 (47.14)	24 (33.80)	0.107
Dyskinesia, n (%)^1^	15 (10.64)	8 (11.43)	7 (9.86)	0.762
Freezing of gait, n (%)^1^	48 (34.04)	28 (40)	20 (28.17)	0.138
MMSE score^3^	26.84 ± 3.03	26.40 ± 3.29	27.27 ± 2.70	0.128
HAMD‐24 score^3^	9.57 ± 7.23	9.86 ± 6.89	9.30 ± 7.59	0.422
Depression, n (%)^1^	83 (58.87)	43 (61.43)	40 (56.34)	0.539
EDS, n (%)^1^	37 (26.24)	19 (27.14)	18 (25.35)	0.658
PDSS score^3^	118.83 ± 20.22	118.04 ± 21.15	119.61 ± 19.38	0.866
RBD, n (%)^1^	74 (52.48)	38 (54.29)	36 (50.70)	0.670
Fatigue, n (%)^1^	31 (21.99)	19 (27.14)	12 (16.90)	0.142
NMS score^3^	34.18 ± 25.75	37.63 ± 27.48	30.77 ± 23.62	0.115
PDQ‐39 score^3^	21.30 ± 17.73	24.07 ± 19.81	18.58 ± 15.05	0.123
WMH Fazekas scale^3^	2.16 ± 1.29	2.90 ± 1.31	1.42 ± 0.75	<0.001*
Short‐form LARS^3^	−6.30 ± 6.10	−6.16 ± 6.86	−6.65 ± 4.97	0.628

Abbreviations: PD, Parkinson’s disease; LED, Levodopa Equivalent Dose; DA, Dopamine agonists; MAO‐B, Monoamine oxidase‐B; UPDRS, Unified Parkinson’s Disease Rating Scale; MMSE, Mini‐Mental State Examination; HAMD, Hamilton Depression Scale; EDS, excessive daytime sleepiness; PDSS, Parkinson’s Disease Sleep Scale; RBD, Rapid Eye Movement Sleep Behavior Disorder; ICRDs, Impulse control and related disorders; NMS, non‐motor symptom; PDQ‐39, 39‐item Parkinson’s Disease Questionnaire; WMH, white matter hyperintensities; LARS, Lille Apathy Rating Scale.

^1^ Chi‐squared tests for categorical variables. Values are expressed as a number (percentage).

^2^ Student t tests for continuous variables with a parametric distribution.

^3^ Mann‐Whitney U tests for continuous variables with a nonparametric distribution.

* Statistically significant (*P* < 0.05).

### Independent association between baseline WMH severity and apathy progression

The linear regression model demonstrated that △short‐form LARS was positively correlated with baseline WMH Fazekas scales (ϐ = 0.959, *P* < 0.001). With increasing WMH severity, there was a faster apathy progression in the group showing apathy at baseline (ϐ = 1.158) versus the group that did not (ϐ = 0.924). There was no significant difference in △short‐form LARS between baseline apathy and nonapathy groups as observed in a Mann–Whitney *U* test (*P* = 0.226). The group showing apathy progression had a significantly higher WMH severity compared to those without progression. This association remained significant after additional adjustment for covariates in models 2 and 3 (Table 5). The area under the ROC curve (AUC) for identifying apathy progression was the WMH Fazekas scale (0.831, 95% CI (0.764‐0.897)), followed by the PVH grade (0.800, 95% CI (0.727‐0.873)), and the DWMH grade (0.733, 95% CI (0.652‐0.814)),which demonstrated diagnostic value. The optimal cut‐off level for the total WMH score to discriminate between apathy and nonapathy groups was 2.5. Since the scoring system did not include 0.5, it could be inferred that a score of ≥3 was indicative of a diagnosis of apathy with a sensitivity of 52.9% and a specificity of 100%.

**Table 5:** Logistic regression analysis between WMH severity characteristics and apathy progression according to different models.

Variable	Apathy progression					
	Model 1		Model 2		Model 3	
	OR (95% CI)	*P*‐value	OR (95% CI)	*P*‐value	OR (95% CI)	*P*‐value
WMH Fazekas scale	7.610 (3.285‐17.630)	<0.001*	8.589 (3.396‐21.719)	<0.001*	10.894 (3.940‐30.123)	<0.001*
PVH grade	35.462 (7.596‐165.554)	<0.001*	56.083 (8.974‐350.474)	<0.001*	139.688 (15.823‐1233.231)	<0.001*
DWMH grade	8.031 (3.037‐21.231)	<0.001*	9.024 (3.236‐25.163)	<0.001*	9.863 (3.456‐28.149)	<0.001*

Abbreviations: WMH, white matter hyperintensities; PVH, periventricular hyperintensities; DWMH, deep subcortical white matter hyperintensities; CI, confidence interval; UPDRS, Unified Parkinson’s Disease Rating Scale; MMSE, Mini‐Mental State Examination; PDSS, Parkinson’s Disease Sleep Scale.

Model 1: adjusting for gender, age, age at PD onset, disease duration, and educational level.

Model 2: adjusting for gender, age, age at PD onset, disease duration, educational level, smoking, alcohol, initial presentation of motor symptoms, and total levodopa equivalent dose.

Model 3: adjusting for gender, age, age at PD onset, disease duration, educational level, smoking, alcohol, initial presentation of motor symptoms, and total levodopa equivalent dose, Hoehn and Yahr stage, UPDRS part III, PDSS score.

*Statistically significant (*P* < 0.05).

## Discussion

Apathy is a common nonmotor feature of PD that can severely affect the quality of life of both patients and caregivers.^3^, ^14^, ^15^, ^16^ However, risk factors for and the pathogenesis of apathy remains unknown. In addition, because apathy and depression have similar clinical manifestations, there is no effective indicator to perform a differential diagnosis. In this study, we evaluated the prevalence of and the clinical risk factors for apathy in a prospective longitudinal study. Our study provides a more granular and clinically relevant understanding of the course and progression of apathy in PD patients compared to previous studies. Moreover, we found that WMH screening tests may represent a useful tool for early apathy screening, which can be used to detect the occurrence and monitor the progression of apathy. Such a tool would be extremely useful for differentiating apathy from depression to facilitate better clinical management. We also discovered that cerebral small vessel disease could be involved in the pathology of apathy in PD, which might serve as a therapeutic target in the future.

Our study included 141 patients with PD who underwent head MRI scans and a follow‐up period of 2.5 years. We recorded the clinical characteristics of the enrolled patients at baseline and the change in apathy scores after 2.5 years of follow‐up. All patients underwent brain magnetic resonance imaging and WMH screening tests at baseline. We found that 48.23% of PD patients suffered from apathy at baseline in this population, which is comparable to rates reported in Caucasian persons, although higher than typically reported for Chinese persons.^2^, ^17^ This might be due to the selection criteria in the Hui Liu et al. study, which enrolled Chinese patients who were treatment‐naïve and had mild motor and nonmotor symptoms. Despite a diagnosis of apathy, which included notifying the patient and the caregiver, most of the patients with apathy continued to be symptomatic 2.5 years later; 76 of 141 PD patients (53.90%) still were positive for apathy during follow‐up. Linear regression indicated that patients with apathy at baseline had a faster apathy progression, which steadily progressed as WMH severity increased. Therefore, we advocate that physicians should pay careful attention to apathy patients with a higher baseline WMH severity.

Our study also confirmed that several previously identified factors were correlated with apathy in PD, including male sex, deteriorating motor symptoms, depression, severe nonmotor symptoms, and a low quality of life.^18^, ^19^ In addition, there was no significant difference in the cognitive performance and medication history between patients with and without apathy. There was no statistical difference between the apathy and nonapathy groups in terms of medication use, not even for levodopa or a dopamine receptor agonist (Table 1). However, levodopa treatment was reported to be an influencing factor in the development and progression of apathy in other studies. Therefore, whether drug treatment is a confounding factor in the development of PD‐associated apathy merits further study. Nonetheless, we found that apathy symptoms were associated with white matter changes, which is consistent with the results of a DTI study.^20^ In our study, WMH severity demonstrated an odds ratio of 2.223 for the association with apathy, adjusting for gender, age, age at PD onset, disease duration, and educational level. We did not find any correlations between WMH location and apathy. Here, the AUC of WMH severity that was most ideal for differentiating apathy from nonapathy was 0.722, indicative of diagnostic power. Our linear regression model demonstrated that the WMH score was linearly related to apathy performance at baseline(ϐ = 1.828, *P* < 0.001).Therefore, WMH severity may be a suitable marker for diagnosing and monitoring apathy. We recommend its regular use in screening for apathy in PD patients that do not exhibit signs of dementia.

Interestingly, WMH severity was significantly different between the apathy and the nonapathy group, but not between the depression and nondepression groups. This was in line with the findings of Hollocks et al.^21^ who reported that white matter microstructural changes were associated with apathy, but not directly related to depressive symptoms in patients with cerebral small vessel disease. Although the neurobiology of apathy in PD is complex and likely involves several different pathophysiological mechanisms, our results indicate that apathy and not depression is associated with white matter hyperintensities. These findings suggest that apathy is tied to organic factors, especially cerebral small vessel factors, while depression is not.

A cross‐sectional study cannot determine if WMH is a predictor for the progression of apathy. Therefore, we followed patients for 2.5 years. We found that patients with apathy at baseline showed a faster progression, which was more serious among patients with WMH. Moreover, WMH is an independent factor associated with the progression of apathy. WMH can effectively predict the progression of apathy. In our study, the AUC of WMH severity for predicting apathy progression was 0.831, indicative of diagnostic power. Therefore, our study revealed an association between apathy progression and WMH in PD patients, which, to our knowledge, has not been reported before. In a multivariate analysis, a positive correlation between higher apathy scores and more severe WMH was noted in our patients. These finding suggests that apathy is not merely a functional symptom, but implies structural pathological changes, specifically that white matter changes may be the structural basis for apathy. These results also indicate that the occurrence and progression of apathy should be monitored in PD patients with severe WMH. Cerebral small vessel factors may influence the occurrence of apathy, while white matter changes may represent a therapeutic target for apathy.

The pathogenesis of apathy in Parkinson's disease patients has been extensively investigated using MRI and PET‐CT. The association between apathy and neurotransmitters, including dopamine, serotonin, and acetylcholine, has also been studied by other researchers. Both functional MRI and PET‐CT have utility in predicting the prognosis of apathy in PD patients. However, compared with functional MRI and PET‐CT scans, our study shows that head MRI of WMH, assessed using the FLAIR sequence and evaluated using Fazekas visual scales, have several advantages. Firstly, this technique can be easily and quickly performed in all hospitals, even in developing countries and regions. Secondly, compared to PET scans, it does not require contrast agents and is radiation‐free. Thirdly, compared with functional MRI, its application is wider, and it can be used for patients with dementia. At the same time, it is a faster test and often preferred by patients for ease and comfort. Finally, it has a high sensitivity and specificity in predicting the prognosis of apathy in PD patients, consistent with other functional MRI and PET‐CT research. Therefore, it has important value in the clinical diagnosis and treatment of PD with apathy.

However, our study does come with a few limitations. Firstly, our study did not include premorbid or de novo, untreated PD patients. Therefore, the presence of WMH cannot be assumed to be a risk factor for the development of apathy, and we cannot deduce apathy results from cerebral small vessel disease. We did not follow‐up patients on imaging of white matter lesions. Therefore, it cannot be deduced whether the occurrence of apathy will lead to an increase in white matter lesions. Secondly, PD patients with severe disease were more likely to decline participation. Hence, we might have underestimated the prevalence of apathy. Thirdly, we used the short‐LARS scale and WMH Fazekas scale for apathy diagnosis and WMH assessment, which cannot accurately ascertain the impact of WMH on apathy status. Another drawback is that all subjects were recruited from a single movement disorder center.

From a clinical perspective, apathy is common in PD and is associated with a poor quality of life. WMH screening tests could be an effective tool for the differential diagnosis of apathy and depression. Moreover, WMH screening tests may provide a useful tool for the early identification apathy and could also be used to follow its progression. Our results also suggest that cerebral small vessel disease may be at least part of the cause of apathy in PD and may represent a future therapeutic target.

## Conflict of Interest

The authors report that they do not have any conflicts of interest.

## Author Contributions

Yu Zhang contributed to design and conceptualization of the study and drafting and revision of the manuscript. Guo yong Zhang contributed to data collection and analysis and helped to draft and revise the manuscript. Zi en Zhang analyzed and interpreted data. An qi He collected and analyzed data. Jin Gan analyzed and interpreted data. Zhen guo Liu helped to revise the manuscript and supervised the study.

## Supporting information


**Supplementary Materia S1**. Detailed clinical characteristics of all PD patients completed all baseline evaluations.
