# Supplementary material for: White matter hyperintensities: a marker for apathy in Parkinson’s disease without dementia?
Source: Ann Clin Transl Neurol. 2020 Aug 28;7(9):1692–701. doi: 10.1002/acn3.51159 (PMC7480903; doi:10.1002/acn3.51159)
Supplement: Supplementary file 1 — Supplementary Materia S1. Detailed clinical characteristics of all PD patients completed all baseline evaluations. [file ACN3-7-1692-s001.docx]

| Variable | Total  n = 253 | Apathy  n = 114 | Non-apathy  n = 139 | *P*-value |
| --- | --- | --- | --- | --- |
| Male sex, n (%) ^a^ | 133（52.57%） | 66（57.89%） | 67（48.20%） | 0.124 |
| Age (years) ^b^ | 68.27 ± 7.69 | 69.05 ± 7.93 | 67.63 ± 7.45 | 0.142 |
| Age at PD onset, (years) ^c^ | 61.29 ± 8.76 | 61.85 ± 9.73 | 60.83 ± 7.88 | 0.370 |
| Disease duration (years) ^c^ | 6.91 ± 4.78 | 7.11 ± 5.31 | 6.75 ± 4.31 | 0.548 |
| Educational level, n (%) ^a^ |  |  |  | 0.023* |
| None/first level, n (%) | 9（3.56%） | 6（5.26%） | 3（2.16%） |  |
| Secondary level/high school, n (%) | 97（38.34%） | 50（43.86%） | 47（33.81%） |  |
| University, n (%) | 147（58.10%） | 58（50.88%） | 89（64.03%） |  |
| Smoking, n (%) ^a^ | 37（14.62%） | 21（18.42%） | 16（11.51%） | 0.122 |
| Alcohol, n (%) ^a^ | 33（13.04%） | 13（11.40%） | 20（14.39%） | 0.483 |
| L-dopa medication, n (%) ^a^ | 209（82.61%） | 94（82.46%） | 115（82.73%） | 0.954 |
| L-dopa LED, mg ^c^ | 326.43 ± 253.18 | 331.83 ± 263.07 | 322.01 ± 245.64 | 0.759 |
| DA medication, n (%) ^a^ | 162（64.03%） | 68（60.18%） | 94（67.63%） | 0.220 |
| DA LED, mg ^c^ | 56.78 ± 54.87 | 51.46 ± 54.17 | 61.11 ± 55.24 | 0.166 |
| MAO-B medication, n (%) ^a^ | 69（27.27%） | 27（23.68%） | 42（30.22%） | 0.246 |
| MAO-B LED, mg ^c^ | 30.63 ± 81.38 | 21.05 ± 56.50 | 38.49 ± 96.65 | 0.075 |
| Total LED, mg ^c^ | 435.30 ± 309.03 | 427.82 ± 317.33 | 441.38 ± 303.14 | 0.730 |
| Initial presentation of motor symptoms, n (%) ^a^ |  |  |  | 0.501 |
| Tremor | 167（66.01%） | 70（61.40%） | 97（69.78%） |  |
| Rigid | 30（11.86%） | 14（12.28%） | 16（11.51%） |  |
| Bradykinesia | 40（15.81%） | 21（18.42%） | 19（13.67%） |  |
| Other | 16（6.32%） | 9（7.89%） | 7（5.04%） |  |
| Hoehn and Yahr stage, n (%) ^a^ |  |  |  | <0.001* |
| 1 | 17（6.72%） | 3（2.63%） | 14（10.07%） |  |
| 1.5 | 47（18.58%） | 14（12.28%） | 33（23.74%） |  |
| 2 | 61（24.11%） | 24（21.05%） | 37（26.62%） |  |
| 2.5 | 70（27.67%） | 39（34.21%） | 31（22.30%） |  |
| 3 | 45（17.79%） | 25（21.93%） | 20（14.39%） |  |
| 4-5 | 13（5.14%） | 9（7.89%） | 4（2.88%） |  |
| UPDRS part III ^c^ | 20.53 ± 11.42 | 23.75 ± 11.72 | 17.89 ± 10.49 | <0.001* |
| Wearing off, n (%) ^a^ | 92（36.36%） | 40（35.09%） | 52（37.41%） | 0.702 |
| Dyskinesia, n (%) ^a^ | 28（11.07%） | 13（11.40%） | 15（10.79%） | 0.877 |
| Freezing of gait, n (%) ^a^ | 92（36.36%） | 44（38.60%） | 48（34.53%） | 0.504 |
| MMSE score ^c^ | 25.52 ± 4.67 | 24.98 ± 5.17 | 26.21 ± 3.92 | 0.206 |
| HAMD-24 score ^c^ | 9.56 ± 8.52 | 11.80 ± 8.89 | 6.69 ± 6.38 | 0.003 |
| Depression, n (%) ^a^ | 47（52.81%） | 33（66.00%） | 14（35.90%） | 0.005 |
| EDS, n (%) ^a^ | 25（28.09%） | 14（28.00%） | 11（28.21%） | 0.983 |
| PDSS score ^c^ | 119.52 ± 21.53 | 116.76 ± 23.14 | 123.05 ± 18.98 | 0.173 |
| RBD, n (%) ^a^ | 43（48.31%） | 25（50.00%） | 18（46.15%） | 0.719 |
| ICRDs, n (%) ^a^ | 29（32.58%） | 17（34.00%） | 12（30.77%） | 0.747 |
| Fatigue, n (%) ^a^ | 16（17.98%） | 12（24.00%） | 4（10.26%） | 0.094 |
| NMS score ^c^ | 35.29 ± 28.80 | 42.22 ± 31.40 | 26.41 ± 22.48 | 0.009 |
| PDQ-39 score ^c^ | 23.46 ± 21.30 | 30.64 ± 23.34 | 14.26 ± 13.92 | <0.001* |

Abbreviations: PD, Parkinson’s disease; LED, Levodopa Equivalent Dose; DA, Dopamine agonists; MAO-B, Monoamine oxidase-B; UPDRS, Unified Parkinson’s Disease Rating Scale; MMSE, Mini-Mental State Examination; HAMD, Hamilton Depression Scale; EDS, excessive daytime sleepiness; PDSS, Parkinson’s Disease Sleep Scale; RBD, Rapid Eye Movement Sleep Behavior Disorder; NMS, non-motor symptom; PDQ-39, 39-item Parkinson’s Disease Questionnaire.

^a^ Chi-squared tests for categorical variables. Values are expressed as number (percentage).

^b^ Student t tests for continuous variables with parametric distribution.

^c^ Mann-Whitney U tests for continuous variables with nonparametric distribution.

^*^ Statistically significant (p < 0.05).
